# Correction to “Experimental study of a novel mouse model of tibial shaft fracture combined with blunt chest trauma”

**DOI:** 10.1002/ame2.70319

**Published:** 2026-10-25

Zhang B, Fu T, Han Y, Li G, Wan X, Li Y. Experimental study of a novel mouse model of tibial shaft fracture combined with blunt chest trauma. *Anim Models Exp Med*. 2025;8:696–706. https://doi.org/10.1002/ame2.12379.

In Figure 4A, the images of the lung tissues of the control group and the multiple trauma group were incorrect. During a re‐examination of the original data, we confirmed that these images did not belong to the animal experimental results reported in the manuscript, which was constructed based on data from different batches of animal experiments. Owing to the large number of gross lung anatomical images collected across experimental batches and the absence of an automatic naming mechanism during image collection, an error occurred during manual file organization, which led to the erroneous inclusion of these images in the figure set of the manuscript. We hereby confirm that dedicated gross lung anatomical images from the corresponding independent experimental batch have been obtained, and that the corrected version of Figure 4A will use the correct images. This was an unintentional error during file management, for which we take full responsibility.

The corrected figure 4 is provided below

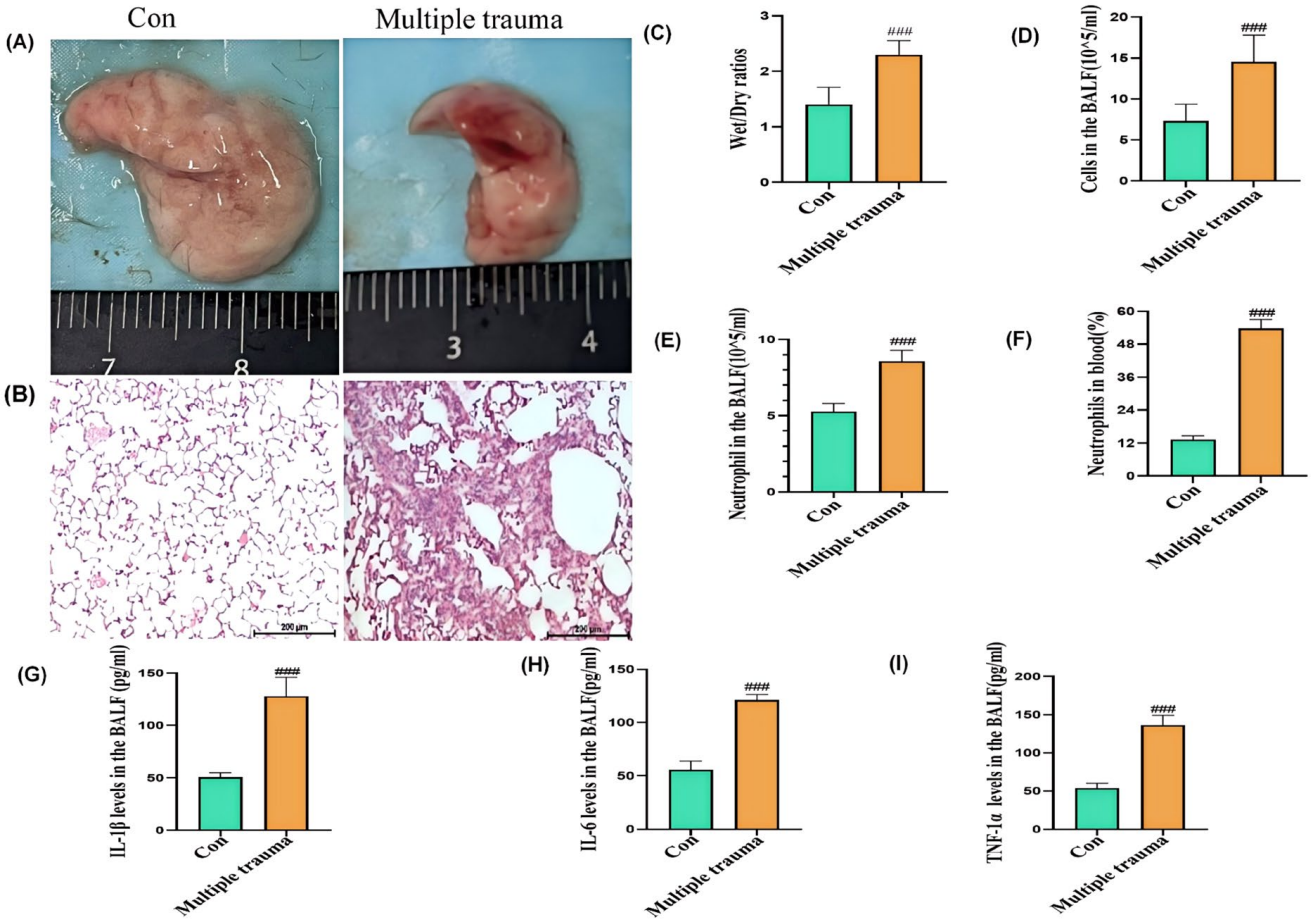

**FIGURE 4** Experimental results of a mouse model of multiple trauma. Morphology of lung tissues collected from the control group and multiple trauma group after the operation (A); hematoxylin and eosin (H&E) staining of the lung tissues of the mice in the multiple trauma group and the control group (B); wet/dry weight ratio of lung tissues in the control group and the experimental group after the operation (C); changes in total cells, total neutrophils, interleukin‐6 (IL‐6), tumor necrosis factor‐1α (TNF‐1α), and IL‐1β in the bronchoalveolar lavage fluid obtained from the control group and the experimental group (D–I) (*n* = 6); ### for *p*< 0.001.

We apologize for this error.

